# Motives for desiring children among individuals of different sexual–romantic orientations: a cross-sectional study

**DOI:** 10.1007/s00404-023-07312-1

**Published:** 2023-12-20

**Authors:** R. Widmer, L. Knabben, N. Bitterlich, M. von Wolff, Petra Stute

**Affiliations:** 1 Department of Internal Medicine at the Checkpoint Zürich, Arud Centre for Addiction Medicine, Schützengasse 31, 8001 Zurich, Switzerland; 2 Department of Obstetrics and Gynecology, University Women’s Hospital, Inselspital, University Hospital of Bern, Friedbühlstrasse 19, 3010 Bern, Switzerland; 3 Medizin & Service GmbH, Boettcherstrasse 10, 09117 Chemnitz, Germany

**Keywords:** Parenting, Fulfilment of the desire to have children, Homosexual, Bisexual, Pansexual

## Abstract

**Purpose:**

Little is known about the reasoning behind the desire to have children in non-heterosexual individuals. This study compares the motives of different sexual–romantic orientations and their preferred ways of fulfilling this desire.

**Methods:**

This was a monocentric cross-sectional study. Subjects were recruited via social media, personal contacts and queer organisations in Switzerland.

An anonymous questionnaire comprised general questions about the participant's background, a validated survey about the desire to have children and additional non-validated questions addressing the impact of sexual–romantic orientation on the desire to have children. The inclusion criteria were adults without children and a completed questionnaire.

**Results:**

Of 837 participants, 642 were included in the study. Four groups of sexual–romantic orientations consisted of more than 35 participants: bisexual–biromantic (*n* = 38), heterosexual–heteroromantic (*n* = 230), homosexual–homoromantic (*n* = 159) and pansexual–panromantic (*n* = 55).

Subgroups with a positive wish for a child rated all motives in the same order and with minimal numeric difference. The most important aspect seemed to be emotional involvement. Non-heterosexual–heteroromantic showed concerns about adverse reactions regarding their wish for a child. All orientations hoped for a biological child.

**Conclusion:**

Our findings about bi-, hetero-, homo- and pansexual people and their motives for a desire to have children agree with the existing literature about hetero, homo and bisexual. The impact of the fear of adverse reaction and discrimination has been discussed before and is supported by our data. We suggest better support before and during the realization of the wish for a child as well as support for non-traditional aspiring parents.

**Supplementary Information:**

The online version contains supplementary material available at 10.1007/s00404-023-07312-1.

## What does this study add to the clinical work

**Table:** 

Individuals of all sexual–romantic orientations have the same motive underlying desire to have children. Health professionals should be aware that 10% of their clients may face difficulties in fulfilling their desire to have children due to their sexual–romantic orientation.	

## Introduction

Having children is widely recognized as a basic human need. Swiss statistics reveal that 90% of childless women and 92% of childless men aged 20–29 express a wish to become parents [1]. However, most studies have focused on heterosexual populations, and the European Society of Human Reproduction and Embryology (ESHRE) reported a lack of research about “non-standard” reproduction, meaning reproduction of same-sex couples, transgender individuals and singles [2].

Recently, the traditional definition of sexual orientation has been extended to include asexuality and pansexuality, alongside hetero-, homo-, and bisexuality [3, 4]. In addition, it has been shown that sexual attraction and romantic attraction can differ from one other [5, 6]meaning that sexual orientation consists of at least two aspects: sexual and romantic attraction.

The most recent data show that 10–25% of the general population does not identify as heterosexual, with varying rates within this range according to age [3, 7]. Within this 10–25%, the proportion of individuals identifying with each of the abovementioned orientations remains unclear, but a national survey conducted in New Zealand provides a basis. Here 2.6–5% of participants reported being homosexual (attracted to the same binary gender), 1.8–3% bisexual (experiencing sexual attraction to both binary genders), 0.3–1% as asexual (experiencing no sexual attraction), and 0.5% as pansexual (experiencing sexual attraction to any gender) [8].

There is limited data available on the proportion of parents who identify as non-heterosexual. In 2018 in Switzerland, 1.2% of all couples were homosexual [1]⁠. In contrast the same study found that, homosexual parents made up only 0.1% of all one-family households with a child younger than 25 years old [9].

Studies focusing on factors driving the desire for parenthood among heterosexuals showed that personal, emotional motives seemed to be most important, identifying statements such as “giving and receiving love” and “founding a family,” as predominant underlying reasons [10, 11].

Previous studies examining motives for desiring children only found minor differences between different sexual orientations. Both heterosexual and homosexual individuals rated the emotional aspects of having children as more important than social recognition or personal and financial constraints [12]. For heterosexual women, parenthood holds significant importance in shaping their identity, while this aspect of parenthood appears to be less crucial for lesbians [13]. Kranz et al. questioned 628 gays on their motives for parenthood and found no difference compared to heterosexuals [14]. In contrast, Goldberg et al. reported differences e.g., the belief among gay men that parenting is psychologically or personally rewarding [15]. Several authors have suggested that fear of stigmatization might impact the desire for children among gay, lesbian, or bisexual individuals [16, 17].

In the rare studies conducted on non-heterosexual populations, only homosexual or bisexual individuals were analyzed. To the best of our knowledge, motives for parenthood in asexual individuals (people with no sexual attraction) or pansexual individuals (people with sexual attraction to any gender) have never been investigated.

This cross-sectional study aims to investigate the desire to have children among individuals of different sexual–romantic orientations, identify motives for parenthood, and preferred methods for fulfilling reproductive needs.

## Methods

### Study and questionnaire design

This monocentric cross-sectional study was performed in July and August 2018. Data were collected through an online questionnaire consisting of three parts, as shown in Supplement 1. In the first section of the questionnaire, baseline characteristics were assessed. The term “sex” was defined as the current allocation to male, female, trans-male, trans-female, or intersex, with “trans” referring to a hormonal transition. The term “gender” was defined as the subjectively perceived identity. The possible options were man, woman, or other. Sexual and romantic orientations were also assessed.

For the second part of the questionnaire, the validated Leipziger “Fragebogen zu Kinderwunschmotiven” [18] was used. This validated survey covered four possible motives for desiring to have children: (1) the desire for emotional stability and finding meaning in life, (2) personal limitations and problems, (3) social recognition and identity building, and (4) insufficient material and social support. Each motive was represented by five statements. Participants rated the relevance of each statement totheir desire to have children using a 5-point Likert scale (1 point = not at all, …, 5 points = very strong), and then the median was calculated. Accordingly, each motive was given a score ranging from 1 to 5 points.

The third part of this study’s questionnaire consisted of ten additional, non-validated questions addressing aspects such as culture, fear of loneliness, impact on a career, genetic diseases, social norms, and the state of the world. The questionnaire was given to individuals with different gender identities and sexual–romantic orientations to assess its comprehensibility. It was adapted where necessary and then approved by the statistician.

### Recruitment

LGBT + organisations and groups in Switzerland and social media were used for recruitment of participants. All individuals aged ≥ 18 could participate. Persons caring for a child in an economic, social or educational context were excluded.

### Statistical evaluation

Data analysis was performed with SPSS 22. Motives for desiring children were analyzed separately for three categories: sexual orientation, romantic orientation, and sexual–romantic orientation. A sexual–romantic orientation was defined as a combination of participants’ sexual orientations (asexual, bisexual, heterosexual, homosexual, pansexual, other) and romantic orientations (aromantic, biromantic, heteroromantic, homoromantic, panromantic, other), resulting in 36 different sexual–romantic orientations. Each sexual–romantic orientation defined a group.

To compare the motives listed in the validated questionnaire (Leipziger Kinderwunschfragebogen), the statement’s ratings ranging from “not important at all” to “very important” were transformed into numeric values of 1–5. The median rating was calculated for each participant. The Kruskal–Wallis test was employed to examine differences between the included groups, and the Mann–Whitney *U* test was applied for pairwise comparisons. Due to the number of groups to be compared the significance for the Mann–Whitney *U* test was *p* = 0.008. The subgroups, “sexual–romantic orientation with a desire to have a child” and “sexual–romantic orientation without a desire to have a child,” were analyzed separately in the same manner. The same tests were also applied to the self-added statements.

To apply the Mann–Whitney *U* test, a minimum of *n* = 20 cases per group was required to demonstrate a significant difference between the groups with an effect size of less than 1.0 (the joint standard deviation is higher than the difference to be observed) with a significance level of 5% and a power of 80%. Taking into account the influence of multiple testing in […] pairwise comparisons of four selected groups, the number of cases had to be *n* > 35. Sufficient subjects were available for statistical analysis in the following groups: bisexual–biromantic, homosexual–homoromantic, pansexual–panromantic, and heterosexual–heteroromantic. Some subgroups with positive or negative desires for a child had *n* < 35 participants.

Fisher's test was used to compare inter-group differences and to correlate baseline characteristics to the desire to have children. Statistical significance was set at *p* < 0.05. Due to a lack of participants, the categories of intersexual people and trans-female (*n* = 1; *n* = 7) and other sexual or romantic orientations (1% each) could not be analysed separately for baseline characteristics.

The comparison of the preferred method to have children per group was done using the Chi-square test and binominal test.

## Results

### Characteristics of the cohort

Out of 837 participants, 641 were eligible for analysis. 196 were excluded due to incomplete questionnaires (*n* = 90), age < 18 (*n* = 64), already being responsible for a child (*n* = 62). Some participants fulfilled several exclusion criteria. The mean age was 27.1 ± 8.6 years. 64.9% of participants reported having the desire to have children. Table 1 presents the characteristics of the cohort.

**Table 1:** The cohort’s characteristics and percentage of participants with a positive wish for a child

Characteristics	Cohort *n* (%)	Positive wish for a child (%)
Total	641	416 (64.9)
Biological sex		
Female	431 (67.2)	281 (67.5)
Men	178 (27.8)	115 (27.6)
Trans-people and intersex	32 (5.0)	20 (4.8)
Gender		
Women	400 (62.4)	267 (64.2)
Men	190 (29.6)	124 (29.8)
Other	51 (8.0)	25 (6)^b^
Sexual orientation		
Asexual	84 (13.1)	25 (6.0)^a^
Bisexual	64 (10.0)	37 (8.9)
Heterosexual	230 (35.9)	193 (46.4)^b^
Homosexual	178 (27.8)	46 (25.5)
Pansexual	78 (12.2)	54 (12.5)
Other	7 (1.1)	3 (0.7)
Romantic orientation		
Aromantic	22 (3.4)	4 (1.0)^a^
Biromantic	58 (9.0)	33 (7.9)
Heteroromantic	277 (43.2)	213 (51.2)^b^
Homoromantic	189 (29.5)	109 (26.2)
Panromantic	88 (13.7)	54 (13.0)
Other	7 (1.1)	3 (0.7)
Sexual–romantic orientation		
Bisexual–biromantic	38 (5.9)	22 (5.3)
Heterosexual–heteroromantic	220 (34.3)	186 (44.7)^b^
Homosexual–homoromantic	159 (24.8)	93 (22.4)
Pansexual–panromantic	55 (8.6)	37 (8.9)
Other (group size < 35)	169 (26.4)	78 (18.8)
Partnership		
No	260 (38.7)	147 (35.3)
Yes, not in civil union or marriage	327 (51.0)	240 (57.7)
Yes, in civil union or marriage	54 (8.4)	22 (5.3)^a^
Education		
None/other	7 (1.1)	1 (0.2)
Obligatory school	26 (4.1)	17 (65.4)
Apprenticeship	100 (15.6)	68 (68)
Higher education without university	281 (43.8)	169 (60.1)
University	227 (35.4)	161 (70.9)
Employment level		
> 89%	234 (36.5)	152 (36.5)
50–89%	112 (17.5)	63 (15.1)^a^
< 50%	106 (16.5)	71 (17.1)
In education	159 (24.8)	111 (26.7)
Other (inc. unemployed)	30 (4.7)	19 (4.6)
Household income/month		
< 5000 CHF	451 (70.4)	304 (73.1)
5–10,000 CHF	147 (22.9)	91 (21.9)
> 10,000 CHF	43 (6.7)	20 (4.8)^a^
Religious community		
Active	63 (10.0)	50 (12.0)^b^
Passive	308 (47.9)	220 (52.9)^b^
No	270 (42.1)	146 (35.1)

^a^The specific characteristic is associated with a significantly higher percentage of participants not wishing to have children a (*p* < 0.05)

^b^The specific characteristic is associated with a significantly higher percentage of participants wishing to have children (*p* < 0.05)

Heterosexual and heteroromantic orientation and active religious affiliation were associated with a higher desire for children, while asexual orientation, aromantic orientation, marriage/civil union, and higher household income (> 10,000 CHF) were negatively associated with a desire for children.

### Motives for a desire to have children

Table 2 presents the motives for or against parental aspiration. All four groups rated either the motive “search for emotional stability and meaning of life” or the motive “inadequate material and social support” the highest. For each motive, significant differences in scoring were observed between at least two orientations.

**Table 2:** Motives for and against the desire to have children between the sexual–romantic orientations

Motives		Mean + SD	Significances
Search for emotional stability and the meaning of life	Bisexual–biromantic	2.8 ± 1.1	Overall *p* < 0.05 Pairwise comparison: bi vs hetero, hetero vs homo, hetero vs pan *p* < 0.008
	Heterosexual–heteroromantic	3.3 ± 0.9	
	Homosexual–homoromantic	2.7 ± 1.0	
	Pansexual–panromantic	2.6 ± 1.1	
Personal limitations and problems	Bisexual–biromantic	2.3 ± 0.7	Overall *p* > 0.05
	Heterosexual–heteroromantic	2.2 ± 1.6	
	Homosexual–homoromantic	2.1 ± 0.7	
	Pansexual–panromantic	2.3 ± 0.6	
Social acceptance and identity	Bisexual–biromantic	1.4 ± 0.5	Overall *p* < 0.05 Pairwise comparison: hetero vs homo *p* < 0.008
	Heterosexual–heteroromantic	1.6 ± 0.5	
	Homosexual–homoromantic	1.4 ± 0.5	
	Pansexual–panromantic	1.5 ± 0.5	
Inadequate material and social support	Bisexual–biromantic	3.0 ± 0.8	Overall *p* < 0.05 Pairwise comparison: hetero vs pan homo vs pan *p* < 0.008
	Heterosexual–heteroromantic	2.7 ± 0.7	
	Homosexual–homoromantic	2.8 ± 0.7	
	Pansexual–panromantic	3.1 ± 0.6	

*p* values mentioned for all significant differences between 2 orientations. Reading example: The group heterosexual–heteroromantic rated the motive “search for emotional stability and meaning of life” significantly higher (*p* < 0.008) than the groups bisexual–biromantic, homosexual–homoromantic and pansexual–panromantic

A separate analysis was done for the subgroups with a positive desire for children. Significant differences between heterosexual and other orientations were found in all four motives. Further details are outlined in Table 3.

**Table 3:** Motives for and against the desire for children within the subgroup with a positive wish for children

Motives		Mean + SD	Significances
Search for emotional stability and the meaning of life	Bisexual-biromantic	3.4 ± 0.9	Overall *p* < 0.05 Pairwise comparison without significance *p* < 0.008
	Heterosexual-heteroromantic	3.5 ± 0.6	
	Homosexual-homoromantic	3.3 ± 0.7	
	Pansexual-panromantic	3.1 ± 0.9	
Personal limitations and problems	Bisexual-biromantic	2.1 ± 0.5	Overall *p* < 0.05 Pairwise comparison: homo vs pan *p* < 0.008
	Heterosexual-heteroromantic	2.1 ± 0.5	
	homosexual-homoromantic	1.9 ± 0.6	
	Pansexual-panromantic	2.2 ± 0.6	
Social acceptance and identity	Bisexual-biromantic	1.4 ± 0.5	Overall *p* < 0.05 Pairwise comparison: hetero vs homo *p* < 0.008
	Heterosexual-heteroromantic	1.6 ± 0.6	
	Homosexual-homoromantic	1.4 ± 0.5	
	Pansexual-panromantic	1.5 ± 0.5	
Inadequate material and social support	Bisexual-biromantic	3.0 ± 0.7	Overall *p* < 0.05 Pairwise comparison: homo vs pan *p* < 0.008
	Heterosexual-heteroromantic	2.6 ± 0.7	
	Homosexual-homoromantic	2.7 ± 0.7	
	Pansexual-panromantic	3.1 ± 0.6	

*p* values mentioned for all significant differences between two groups. Reading example: The group heterosexual-heteroromantic with a positive wish for child rated the motive “social acceptance and identity” significantly higher (*p* < 0.008) than the homosexual-homorimantic subgroup with a positive wish for children

The subgroups without a desire to have children showed no significant differences in the rating of the motives.

### Additional statements

The scores for the ten additional questions (Supplement 1) in the different subgroups with a positive wish for a child were as followed: In bisexual–biromantics, the highest-rated statement was “I would like to recognize the child/children of my partner,” with a score of 3.7, and the lowest was “A child could be a relationship saver,” with a score of 1.1. Among heterosexual–heteroromantic, the statement “I would like to recognize the child/children of my partner” received the highest rating at 3.1 points, whereas the statement “I fear adverse reactions because of my gender or sexual orientation” was rated the lowest at 1.1 points. Homosexual–homoromantic participants rated “I would like to recognize the child/children of my partner” the highest with 4.0 points, while “In my religion, I am expected to have a child” received the lowest rating at 1.1 points. Pansexual–panromantics also rated the statement “I would like to recognize the child/children of my partner” the highest with 3.7 points, and […]”In my religion, I am expected to have a child” was rated the lowest with 1.1 points.

We found several significant differences in the ratings among the subgroups with a positive wish for children. Non-heterosexual–heteroromantic individuals with a positive wish for a child rated the statement “I fear adverse reactions because of my gender or sexual orientation” significantly higher (*p* < 0.05) than heterosexual–heteroromantic individuals. The statement “I do not want to have a child in this world” was rated significantly differently between heterosexual–heteroromantic and homosexual–homoromantic individuals; the former rated the statement lower (*p* < 0.05). Homosexual–homoromantic individuals also rated the statements “I would like to recognize the child/children of my partner” and “fear of consequences for the child or themselves in case of a divorce” significantly higher than heterosexual–heteroromantic individuals. On the other hand, heterosexual–heteroromantic individuals with a positive desire for a child rated the statement “not wanting to be alone in their old age” (*p* < 0.05) significantly higher than homosexual–homoromantic individuals. According to the Kruskal–Wallis test, the overall significance of the statement “a child could save our [sexual/romantic] relationship” differed significantly among the four orientations; however, there was no significant difference in pairwise comparisons.

The only significant difference in the subgroups with no desire to have children was the “fear of negative reactions because of their sex or gender,” where non-heterosexual–heteroromantic individuals rated the statement significantly higher (p < 0.05).

### Preferred realisation of the desire to have a child

Participants across all orientations expressed a preference for biological children (biological child with a partner, egg cell/insemination, co-parenting, or surrogate mother) over non-biological options (adoption or foster child). However, the desire for a biological child with a partner varied significantly. Specifically, 93.6% of heterosexual–heteroromantic participants and 68.2% of bisexual–biromantic participants expressed a wish for a biological child with a partner. Among homosexual–homoromantic people, 32.3% preferred a donation of semen/egg cells, and 31.2% preferred adoption. Pansexual–panromantic individuals favoured adoption (29.7%) or a biological child with a partner (27.0%).

Of the non-heterosexual–heteroromantic individuals, only 5–10% would choose co-parenting as their preferred method to fulfil their desire to have children. A foster child was chosen solely by 8.60% of the homosexual–homoromantic as the preferred option.

## Discussion

### General outcome

The main findings of our study were that 64.9% of participants expressed a positive desire to have children, with significantly higher rates among heterosexual–heteroromantic participants in comparison to other participant groups. Across the various sexual–romantic orientations, the motivation behind the wish for children was consistent, with most participants hoping for a biological child.

### Rate of desire to have children in different sexual orientations

In contrast to the general population, in which up to 92% express a positive desire for children, our cohort showed a lower rate of 64.9%. This discrepancy could be partially explained by the wider age range of our participants (18–72 years) and the lower rate of desire for children among non-heterosexual individuals who were overrepresented in the present study compared to the general population. In line with previous studies, heterosexual–heteroromantic participants had a significantly higher desire for children than bisexual–biromantic and homosexual–homoromantic individuals [19–23].⁠ To the best of our knowledge, there is to date no study on family planning among pansexual–panromantic individuals. Our data showed that 67% had a positive wish for children. This finding goes along with previous data on bisexual individuals claiming an intermediate rate of desire for children (between that of hetero- and homosexuals) for this group which pansexual–panromantic people are as well [20, 24]⁠.

### Motives concerning the desire to have children

Statistically significant differences were found in the rating of the different motives for the wish for children across various sexual–romantic orientations. However, the practical meaning of these differences may be limited since the ratings were performed on a scale from 1 to 5, with maximal numeric differences of 0.4 points whereas the standard deviation was at least the difference if not the double of the difference itself. We believe that the main motives for desiring children were the same across different sexual–romantic orientations. This lack of major differences and the importance of the emotional aspect has been reported in homosexual individuals compared to heterosexual individuals previously [12–15, 25]*.*

The worr from the additional statements revealed the impact of social expectations on family planning in minority groups, as non-heterosexual individuals expressed greater concerns about negative reactions due to their gender or sexual orientation and hesitations about bringing a child into a potentially hostile world. Reasons for this difference may include: the internalisation of queerphobia and anticipated stigma [12, 16, 17, 26], longing for a queer-friendly surrounding [23, 27], or an expression of lower expectations of life [28] need to be discussed.

### Preferred method of achieving the desire to have children

All four orientations expressed a preference for biological children. Awareness of the prevalence of non-heterosexual–heteroromantic people in the general population highlights the need for comprehensive and sympathetic support from the healthcare system, given that a significant percentage would require assistance in reproduction.

Nearly 1/3 of homosexual–homoromantic or pansexual–panromantic individuals with a positive desire for children preferred adoption (either of a completely foreign child or the legal adoption of the partner’s child) over a biological child. Nevertheless, a genetic relationship still seemed to carry importance, which has also been shown in previous studies on surrogacy and should be taken into account when talking about laws regarding fertility and family law [29, 30].

### Further implications

In this study 54.6% of non-heterosexual participants declared a desire to have children, aligning with previous research [19–23]. However, official statistics in Switzerland showed that only 0.1% of households with children under 25 years of age consisted of homosexual couples in 2018. The actual number might be higher due to children from previous relationships living with heterosexual partners. Assuming that 10% of the population identifies as non-heterosexual [3, 7] and at least 50% of them […] desire children, […] this population faces systematic disadvantages in reproduction.

Several factors contribute to these challenges, including financial constraints [19], legal restrictions [21] and discrimination and the absence of role models [27]. In our opinion, this and the previous findings show that non-heterosexuals need more medical and legal support than their heterosexual counterparts. Health professionals should be aware that 10% of their clients may encounter difficulties in fulfilling their desire to have children. This statement is strongly supported by the European Society of Human Reproduction and Embryology, ESHRE, which declared that lack of support for non-conforming couples regarding their wish for children was a violation of their human rights and that the people concerned wished for more support from their doctors [2, 31]. Additionally, specific support and changes in society for non-normative circumstances are needed [31]

In Switzerland, artificial insemination in married homosexual couples has been legal since July 2022 (Art. 9 g Abs. 2 SchlT ZGB). The impact of this change requires ongoing observation.

## Limitations and strengths

The primary limitation of our study was the small size of certain subgroups that were on the verge of being statistically meaningful using a non-parametrical Mann–Whitney-*U*-Test (e.g., bisexual–biromantic individuals with a desire for children, *n* = 22). Consequently, asexual and aromantic individuals could not be included in detailed analyses. Another limitation was the ten additional statements that were not previously validated in a previous study. However, our study stands as the first to analyze the reproductive needs of pansexual individuals and to consider romantic orientation.

### Supplementary Information

Below is the link to the electronic supplementary material.

Supplementary file1 (DOCX 22 KB)

## Data Availability

Data is available on request.
