# Supplementary material for: Motives for desiring children among individuals of different sexual–romantic orientations: a cross-sectional study
Source: Arch Gynecol Obstet. 2023 Dec 20;309(2):707–14. doi: 10.1007/s00404-023-07312-1 (PMC10808403; doi:10.1007/s00404-023-07312-1)
Supplement: Supplementary file 1 — Supplementary file1 (DOCX 22 KB) [file 404_2023_7312_MOESM1_ESM.docx]

**Title**

Motives for desiring children among individuals of different sexual-romantic orientations: A cross-sectional study

**Author names and affiliations**

R Widmer^a^, L Knabben, M.D.^b^, N Bitterlich, Ph.D.^c^, M von Wolff, M.D. ^b^, P Stute, M.D. ^b^

^a^ Department of Internal Medicine at the Checkpoint Zürich, Arud centre for addiction medicine, Schütezngasse 31, 8001 Zürich, Switzerland.

^b^ Department of Obstetrics and Gynecology, lnselspital, Bern University Hospital, University of Bern, Theodor-Kocher-Haus, Friedbühlstrasse 19, 3010 Bern, Switzerland.

^c^ Medizin & Service GmbH, Boettcherstrasse 10, 09117 Chemnitz, Germany.

**Corresponding author**

Professor Dr. med. Petra Stute, M.D.

Gynecologic Endocrinology and Reproductive Medicine, University Women's Hospital

Theodor-Kocher-Haus, Friedbühlstrasse 19, 3010 Bern, Switzerland

E-mail: [petra.stute@insel.ch](mailto:petra.stute@insel.ch)

## **Supplement 1 – Whole questionnaire**

1. Which biological sex (English: sex) were you assigned at birth? (Usually written on your identity card as f or m)

- female
- male
- intersexual

2. Are you currently taking hormone treatment? (Hormone treatment for gender reassignment in trans people / non-binary. Hormone treatment for gender reassignment or normalisation in intersex people.)

- Yes, female hormones
- Yes, male hormones
- No

3. Which gender identity do you have? (Which identity do you feel like?)

- Woman
- Man

4. Which gender role do you live in everyday life?

- Woman
- Man
- Other, namely:

5. How old are you?

- Please select:

6. What is your sexual orientation?

- Heterosexual (you feel the need to be physically sexually active with the other binary gender (man/woman).
- Homosexual (you feel the need to be sexually active with the same sex/gender)
- Bisexual (you don't care if you are sexually active with a man or a woman)
- Pansexual (it does not matter to you which sex/gender your sexual partner is)
- Asexual (you do not feel the need to be sexually active with anyone)
- Other sexual orientation

7. What is your romantic orientation? (Usually refers to the emotional-social aspect of a relationship)

- Heteroromantic (you fall in love with another sex/gender)
- Homoromantic (you fall in love with the same sex/gender)
- Biromantic (you fall in love with both men and women)
- Panromantic (you fall in love regardless of gender)
- Aromantic (you do not fall in love with anyone)
- Other romantic orientations

8. Are you currently in a partnership? (The term homo/hetero here refers to the legal sex. I.e. the one on your passport.)

- No
- Yes, homosexual relationship, not married
- Yes, heterosexual relationship, not married
- Yes, other relationship, not married
- Yes, registered homosexual/homoromantic partnership
- Yes, registered heterosexual/heteroromantic partnership
- Yes, registered partnership (neither homosexual nor heterosexual/romantic)
- Yes, married
- other

9. Do you have one or more children? (This does not only include biological children, but any children for whom you are responsible either financially or educationally.)

- yes
- no

10. What is your highest schooling degree?

- None
- Compulsory school
- “Attestlehre” (=apprenticeship with a lower degree)
- Apprenticeship
- “Berufsmaturität” (=additional degree after a apprenticeship that allows the attendance at certain colleges but not university)
- “Höhere Fachschule / Fachhochschule” (=tertiary school that is no university)
- Matura (= allows holder to attend an university or any lower tertiary schooling)
- Bachelor at university
- Master at university
- Doctorate
- other

11. Have you ever been employed?

- Never
- Self-employed
- Employed
- Househusband / Housewife / Houseperson

12. You are currently working … (Multiple selections possible)

- Full-time (90% and more)
- Part-time (50- 89%)
- Part-time (0.1-50%)
- Military/civilian service
- Parental leave
- Retired
- In education
- Not at all (unemployed)

13. Do you live with your partner?

- yes
- no

14. What is your personal household income per month, roughly estimated?

- < 5'000 CHF
- 5'000 - 10'000 CHF
- 10'000 CHF

15. Do you belong to a religious community? (e.g. the Catholic Church, Islam...)

- Yes, active (religious, church attendance, etc.)
- Yes, passive (only on paper)
- No

16. In which environment did you grow up? (Multiple choice possible)

- My parents were married
- My parents were in a partnership
- My parents were divorced
- I grew up with a guardian
- I was half-orphan
- I was an orphan
- Other

17. Do you have any siblings?

- yes
- no

18. Do you have a desire to have children? During the time when you were actively thinking about having children, did you have one? Please select the option that is personal number 1.

- No
- Yes, I would like to have a biological child with my partner (self-conceived & carried to term).
- Yes, a biological child but with the help of sperm or egg donation
- Yes, adoption
- Yes, co-parenting (raising a child (or children) together without a relationship)
- Yes, surrogate mother
- Yes, foster child

19 In the previous question, you indicated that you would like to have a child and your number 1 indicated how you would like to have one. If you are seriously considering more than one option, you can indicate them including your number 1. If only one option can be considered for you, tick only this one.

- I would like to have a biological child with my partner (self-conceived & carried to term)
- a biological child but with the help of sperm or egg donation
- adoption
- co-parenting (raising a child (or children) together without a relationship)
- Surrogate mother
- Foster child

For the following statements, mark the extent to which this statement influences your personal motives for wanting to have a child.

In the following questions, the term "own child" refers not only to a biological child but also to any child for whom you would be would have to provide financially or educationally.

Note: The possible answers for questions 20 – 39 were: not at all, barely, partially, considerably and strongly

20. A child gives my life its true meaning

21. I do not want to have a child of my own so that I do not have to listen to reproaches about a wrong upbringing later on

22. With a child I can prove my fertility.

23. There is a lack of the necessary state support for families with children.

24. With a child, I create new life.

25. I am afraid that having my own child would change my sexual relationship with my partner.

26. A child is necessary for me to be recognised as an adult.

27. Having a child is a handicap in our society.

28. I want to experience the development of my own child.

29. With a child of my own, my partner and I no longer have enough time for each other.

30. A successful person also includes children.

31. There are too few daycare facilities for children.

32. A child makes me feel like I have a real home.

33. I am too impatient to raise a child on my own.

34. Having a child gives me a higher status in our society.

35. I can enjoy my free time better without a child.

36. For me, there is no substitute for the love of one’s own child.

37. With a child of my own, I would not be able to maintain my friendships in the same way as before.

38. For me, childlessness means social devaluation.

39. A child costs time. I have to give up many other things I like to do.

40. I wish for an heir for the family.

41. In my religion, I am expected to have a child.

42. A child could save a relationship.

43. I do not want to be alone in my old age.

44. In our society, a child would be too big of an obstacle to my career.

45. I am afraid of divorce and the consequences or my child and me.

46. I am afraid of hereditary diseases

47. I fear adverse reactions because of my gender or sexual orientation.

48. I do not want to put a child in this world.

49. I would like to recognise the child/children of my partner.

Note

- Questions 20-39 are out of the validated questionnaire “Leipziger Fragebogen zu Kinderwunschmotiven” by E. Brähler, Y. Stöbel-Richter and J. Schumacher.
- Statements 20, 24, 28, 32 and 36 together are the motive desire for emotional stability and finding meaning.
- Statements 21, 25, 29, 33 and 37 together are the motive personal limitations and problems.
- Statements 22, 26, 30, 34 and 38 together are the motive social recognition and identity building.
- Statements 23, 27, 31, 35 and 39 together are the motive insufficient material and social support.
- The question 40-49 were created by the authors.
